# Impact of Universal Health Insurance Coverage on Hypertension Management: A Cross-National Study in the United States and England

**DOI:** 10.1371/journal.pone.0083705

**Published:** 2014-01-08

**Authors:** Andrew R. H. Dalton, Eszter P. Vamos, Matthew J. Harris, Gopalakrishnan Netuveli, Robert M. Wachter, Azeem Majeed, Christopher Millett

**Affiliations:** 1 Department of Primary Care Health Sciences, University of Oxford, Oxford, England; 2 Department of Primary Care and Public Health, Imperial College London, London, England; 3 Institute for Health and Human Development, University of East London, London, England; 4 Division of Hospital Medicine, Department of Medicine, University of California San Francisco, San Francisco, California, United States of America; INRCA, Italy

## Abstract

**Background:**

The Patient Protection and Affordable Care Act (ACA) galvanised debate in the United States (US) over universal health coverage. Comparison with countries providing universal coverage may illustrate whether the ACA can improve health outcomes and reduce disparities. We aimed to compare quality and disparities in hypertension management by socio-economic position in the US and England, the latter of which has universal health care.

**Method:**

We used data from the Health and Retirement Survey in the US, and the English Longitudinal Study for Aging from England, including non-Hispanic White respondents aged 50–64 years (US market-based v NHS) and >65 years (US-Medicare v NHS) with diagnosed hypertension. We compared blood pressure control to clinical guideline (140/90 mmHg) and audit (150/90 mmHg) targets; mean systolic and diastolic blood pressure and antihypertensive prescribing, and disparities in each by educational attainment, income and wealth, using regression models.

**Results:**

There were no significant differences in aggregate achievement of clinical targets aged 50 to 65 years (US market-based vs. NHS- 62.3% vs. 61.3% [p = 0.835]). There was, however, greater control in the US in patients aged 65 years and over (US Medicare vs. NHS- 53.5% vs. 58.2% [p = 0.043]). England had no significant socioeconomic disparity in blood pressure control (60.9% vs. 63.5% [p = 0.588], high and low wealth aged ≥65 years). The US had socioeconomic differences in the 50–64 years group (71.7% vs. 55.2% [p = 0.003], high and low wealth); these were attenuated but not abolished in Medicare beneficiaries.

**Conclusion:**

Moves towards universal health coverage in the US may reduce disparities in hypertension management. The current situation, providing universal coverage for residents aged 65 years and over, may not be sufficient for equality in care.

## Introduction

Given the growing global burden of chronic diseases, effective and equitable management of diseases such as hypertension is a core requirement of any health system [1]. Despite population level reductions in blood pressure and improvements in anti-hypertensive prescribing [2], [3],_ENREF_2 suboptimal hypertension management – most notably insufficient blood pressure control – persists in many countries, including the United States (US) and England. One marker of this quality gap across all countries is within-country disparities in hypertension management, which are likely due to population, health care provider and health system characteristics [4].

Cross-country analysis can examine relationships between health system structures and outcomes [5], [6]. England and the US have four important differences that may influence hypertension management. Firstly, the US has a more specialised health service (higher ratio of specialists to generalists; more ambulatory care delivered by specialists) whereas the National Health Service (NHS) emphasizes generalist primary care, with restricted access to specialists through gate-keeping. Strong primary care can improve health outcomes [7], [8] and may reduce inequalities [7], especially in conditions requiring longitudinal care such as hypertension [9]. Second, as a single payer system, the NHS arguably has clearer lines of accountability and more centralized coordination of quality improvement (though the more pluralistic US system may be more innovative). Thirdly, the US has considerably higher health care spending than England; although this may be considered a result of other structural differences [10]. In 2009 the US spent 17.4 percent of its Gross Domestic Product on health care, compared with 9.8 percent in the United Kingdom (UK). This may provide resources for higher quality care, although the relationship between health care spending and quality remains unclear [10].

Finally, England offers universal health coverage with care free at the point of delivery. Medication in England costs a standard prescription charge (£7.10 (∼$13.90) in April 2008), although exemptions mean that approximately 60 percent of the population, and 90 percent of items dispensed are free of charge [11]. Exempt groups include people aged 60 years and over, people on income support, or those with certain chronic health conditions – although not hypertension. The US has universal coverage for people aged 65 years and over, through Medicare. Co-payments for medications, however, remain high, despite Medicare's “Part D” drug prescription benefit program [12]. Health coverage for younger US patients is more variable, with a high proportion without insurance or under-insured [4]. Having survived legal contests over the “individual mandate” and given the outcome of the 2012 presidential election, the Patient Protection and Affordable Care Act (ACA) will partly address this. It will expand Medicaid eligibility and enhance individual access to private health insurance, although the former may be limited by the power of individual states not to implement changes [13].

We aim to determine whether health system differences between the US and England influence the quality of hypertension management and disparities across socio-economic position (SEP), characterised by income, wealth and education [14]. By stratifying respondents into one group younger than 65 years and the other older (i.e. the eligibility criteria for Medicare), we compare hypertension management in the NHS both with part of the US health care system with universal (Medicare) coverage and with the market-based system for those under 65. The latter will see coverage expanded as part of the ACA.

## Materials and Methods

### Data Sources

We analysed data from two national longitudinal surveys, the English Longitudinal Study of Aging (ELSA), and the Health and Retirement Survey (HRS) from the US. These provide comparable data on aging, with similar sampling techniques and content [5]. Both are population-based surveys presenting information on health, physical and mental functioning, demographic factors, behaviors and SEP in non-institutionalized individuals aged 50 years and over.

ELSA, a biennial survey, began in 2002, sampling respondents aged 50 years and over [15]. A refreshment sample, aged 50 to 53 years, was added in 2006 to boost representation of younger age groups. Interviews are carried out at respondents' homes, with visiting nurses collecting biomedical data during alternate waves. The HRS started in 1991 [16], with biennial follow-up since 1992 and subsequent refreshment samples. The HRS has maintained a sample of more than 20,000 respondents, and is conducted using both telephone and face-to-face interviews – dependent on the survey wave and module of questions. Further details of both surveys can be found elsewhere [15], [16].

### Study sample

We used data from Wave 4 of ELSA, conducted during 2008 and 2009, which contained 11,050 respondents (response rate 74.3%). We included respondents aged 50 years and over, with valid blood pressure readings (the mean of two or more readings on the same day― these are considered valid for both surveys) from the survey nurse visit (n = 7,982). To control for differences in ethnic mix between samples, and ensure that we compared differences between health systems – not biological differences in control between samples, we restricted analyses to non-Hispanic Whites (excluding 225 from ELSA). We exclude respondents with missing SEP (n = 2,795) and clinical data (body mass index (BMI), co-morbidities and smoking status; n = 900 – latter two not mutually exclusive), leaving 4,910 respondents.

For comparison we used data from Wave 9 of the HRS, administered in 2008, with 17,217 respondents (response rate 88.6%). We include respondents aged 50 years and over, with valid blood pressure readings (mean of two of more – n = 8,273). We excluded 3,049 non-white and Hispanic respondents, 304 with no BMI record (there were no missing SEP data), leaving 4,920 respondents.

From the samples defined above, we include respondents with a self-reported physician's diagnosis of hypertension, leaving 2,318 ELSA and 2,855 HRS respondents. Both surveys included a question similar to “*Has a doctor ever told you that you have hypertension*?” Though this method may underestimate hypertension rates compared with measures based on actual blood pressure values [5], we felt it a more relevant definition for our study, which relates to the management of patients with a known diagnosis – further there is no evidence of inequalities in reporting-bias [15].

### Predictor variables – measures of SEP

We used three measures of SEP that are comparable between countries: education, household income and wealth. We divided education into three groups defined using years of continuous education; in England 0–11 years, 12–13 and ≥14; in the USA 0–12, 13–15 and ≥16 years. These cut-offs are widely used [5], [6], producing similar education distributions across countries. We used total household income, and total household wealth (savings, net stock value, mutual funds, bonds, real estate value, own business share, owned cars minus liabilities), adjusted for household size (the square root of household size), and producing groups separated by tertiles within each country.

### Outcome variables

We used mean systolic and diastolic blood pressure (from two or more measures), defined controlled hypertension as blood pressure lower than 140/90 mmHg, in line with clinical guidelines [17], [18] and measured prescribing of anti-hypertensive agents based on self-report. In addition to the guideline-derived target, we assessed control (in both countries) based on the UK's audit target of 150/90 mmHg. This defines control in England's Quality and Outcome Framework (QOF), which provides pay-for-performance remuneration to primary care practices for the management of chronic disease, including hypertension, therefore is an important UK threshold for control [19].

### Covariates

We controlled for the following potentially confounding variables: age and sex of respondents; smoking status (current/non-smoker); BMI; and concordant vascular co-morbidities. All except BMI were self-reported, which was recorded by study nurses. We defined the latter as the sum of patient reported doctor diagnoses out of coronary heart disease, stroke/transient ischaemic attack, diabetes or chronic kidney disease.

### Statistical Methods

We conducted analyses separately in the groups aged 50 to 64 years and 65 years and over. We present the prevalence of hypertension, standardized for age and sex, along with summaries of covariates across indicators of SEP (education, income and wealth), in patients with hypertension in each country, testing for statistical significance.

In patients with hypertension, we modelled blood pressure control (to clinical and audit targets) and anti-hypertensive medication using logistic regression [20]; and systolic and diastolic blood pressure using linear regression. We modelled outcomes separately in each country, age group and individual measure of SEP; adjusting for age, sex, smoking status, vascular co-morbidity and BMI. In the 50 to 64 years age group in the US, we present results in the whole population and the insured group alone. We defined the insured group as those reporting any health insurance coverage (private, Medicaid or other health care plan) at the time of interview. We present adjusted outcomes after recombination of model coefficient [20], presenting the overal control in each country and in each SEP group. We test statistical significance in global control between countries; between SEP groups within each country, comparing medium and high, with lowest group for each indicator; and in equivalent SEP groups between countries. We derived standard errors using the delta method for all adjusted coefficiants [21]; when unable to estimate variance directly, this allows accurate estimation [21]. We use valid survey weights in all analyses (variables *w4nurwt* in ELSA and *lwgtr* in the HRS), which we conducted using Stata 11.2SE.

## Results

The unadjusted prevalence of hypertension in England and the US was 38.2% and 45.1% in respondents aged 50–64 years and 52.9% and 63.6% in those aged >65 years, respectively. There are significant disparities in prevalence, especially by education and wealth, which were greatest in US respondents aged 50–64 years. The characteristics of respondents with hypertension are presented in table 1. In both age groups, there was higher mean BMI and prevalence of vascular co-morbidity in the US. There are marked gradients in smoking in both countries, with a higher prevalence in lowest SEP groups and, in younger patients, higher BMI in lowest SEP groups. There are significant SEP disparities in insurance coverage in US respondents aged 50–64 years.

**Table 1:** Risk factors and characteristics in hypertensive patients aged greater than 50 years in England and the United States.

	England				United States			
	Years of education							
	Low	Medium	High	Total	Low	Medium	High	Total
*Age 50 to 64 years*								
N hypertensive¤	451	63	212	726	340	164	164	668
Prevalence, % (SE)	35.9 (2.2)	30.2 (3.5)	30.1 (2.2)	33.4 (1.8)	47.3 (2.4)‡	43 (2.9)‡	33.4 (2.6)	41.7 (1.7)‡
Sex (% Female, SE)¤	54.0 (2.4)	39.9 (6.1)	40.0 (3.3)	49.3 (1.9)	48.5 (3.8)	38.1 (5.1)	35.6 (5.3)	42.8 (2.7)
Age, mean (SE)¤	60.3 (0.1)	59.7 (0.3)	59.9 (0.2)	60.1 (0.1)	59.2 (0.2)	59.1 (0.3)	59.2 (0.3)	59.2 (0.2)
Current smoker, % (SE)	20.5 (2.0)	12.6 (4.4)	8.2 (2.2)	16.7 (1.5)	25.0 (2.7)	19.3 (3.5)	11.6 (3.0)	20.0 (1.8)
BMI mean (SE)	30.6 (0.3)	29.9 (0.7)	29.3 (0.3)	30.2 (0.2)	31.5 (0.5)	31.9 (0.7)‡	30.7 (0.7)	31.4 (0.3)‡
>1 Vascular Co Morb, % (SE)	17.3 (1.8)	20.5 (5.2)	12.1 (2.2)	16.2 (1.4)	38.4 (3.7)‡	27.9 (4.9)‡	20.4 (4.8)‡	31.4 (2.5)‡
Insurance coverage, % (SE)					86.0 (2.6)	94.2 (2.4)	96.0 (2.5)	90.5 (1.6)
*Age*≥*65 years*								
N hypertensive¤	1195	68	329	1592	1344	416	427	2187
Prevalence, % (SE)	55.0 (1.1)	46.1 (4.1)	48.4 (1.9)	53.4 (0.9)	65.2 (1.2)‡	61.7 (2.1)‡	58.9 (2.0)‡	63.1 (0.9)‡
Sex (% Female, SE)¤	61.3 (1.4)	43.3 (6.0)	39.3 (2.7)	57.2 (1.3)	54.3 (1.9)	51.6 (3.4)	33.3 (3.1)	49.7 (1.5)
Age, mean (SE)¤	76.6 (0.2)	75.3 (1.0)	74.3 (0. 5)	76.2 (0.2)	74.8 (0.3)	73.7 (0.5)	74.0 (0. 5)	74.4 (0.2)
Current smoker, % (SE)	10.3 (0.9)	4.7 (2.7)	5.2 (1.2)	9.3 (0.8)	11.1 (0.9)	9.6 (1.8)	5.0 (1.2)	9.6 (0.7)
BMI mean (SE)	28.9 (0.2)	29.2 (0.8)	28.2 (0.3)	28.8 (0.1)	29.8 (0.2)‡	29.8 (0.5)	29.2 (0.3)‡	29.7 (0.2)‡
>1 Vascular Co Morb, % (SE)	27.5 (1.3)	33.4 (5.7)	24.9 (2.4)	27.3 (1.1)	41.1 (1.9)‡	35.0 (3.2)	31.6 (3.1)‡	38.0 (1.4)‡
	**Income**							
	**Low**	**Medium**	**High**	**Total**	**Low**	**Medium**	**High**	**Total**
*Age 50 to 64 years*								
N hypertensive¤	184	250	292	726	161	196	311	668
Prevalence, % (SE)	37.7 (2.8)	35.2 (2.5)	30.1 (2.0)	33.4 (1.8)	47.8 (3.4)‡	45.2 (3.0)‡	38.1 (2.1)‡	41.7 (1.7)‡
Sex (% Female, SE)¤	51.6 (3.8)	49.7 (3.2)	47.2 (2.9)	49.3 (1.9)	53.1 (5.5)	47.1 (5.1)	34.5 (3.7)	42.8 (2.7)
Age, mean (SE)¤	60.1 (0.2)	60.4 (0.2)	60.0 (0.2)	60.1 (0.1)	59.0 (0.3)	59.2 (0.3)	59.3 (0.2)	59.2 (0.2)
Current smoker, % (SE)	23.7 (3.3)	18.4 (2.6)	10.1 (1.9)	16.7 (1.5)	29.0 (4.1)	24.0 (3.5)	13.6 (2.2)	20.0 (1.8)
BMI mean (SE)	31.2 (0.5)	30.2 (0.4)	29.6 (0.3)	30.2 (0.2)	32.3 (0.7)	31.6 (0.7)‡	30.9 (0.5)	31.4 (0.3)‡
>1 Vascular Co Morb, % (SE)	23.2 (3.2)	16.9 (2.4)	10.5 (1.8)	16.2 (1.4)	53.3 (5.4)‡	24.0 (4.4)‡	23.7 (3.4)‡	31.4 (2.5)‡
Insurance coverage, % (SE)					75.3 (4.7)	94.0 (2.2)	96.8 (1.4)	90.5 (1.6)
*Age*≥*65 years*								
N hypertensive¤	626	633	333	1592	762	856	569	2187
Prevalence, % (SE)	54.8 (1.5)	54.3 (1.5)	49.0 (1.9)	53.4 (0.9)	68.5 (1.6)‡	63.2 (1.4)‡	57.2 (1.7)‡	63.1 (0.9)‡
Sex (% Female, SE)¤	64.8 (1.9)	53.6 (2.0)	47.1 (2.8)	57.2 (1.3)	55.4 (2.6)	50.3 (2.3)	41.6 (2.8)	49.7 (1.5)
Age, mean (SE)¤	77.5 (0.3)	75.9 (0.3)	74.0 (0.4)	76.2 (0.2)	74.9 (0.4)	74.3 (0.3)	73.9 (0.4)	74.4 (0.2)
Current smoker, % (SE)	10.1 (1.2)	9.9 (1.2)	6.0 (1.3)	9.3 (0.8)	12.9 (1.4)	9.5 (1.1)	5.3 (1.0)	9.6 (0.7)
BMI mean (SE)	28.6 (0.2)	28.9 (0.2)	29.0 (0.3)	28.8 (0.1)	29.1 (0.3)‡	30.1 (0.3)‡	29.6 (0.3)	29.7 (0.2)‡
>1 Vascular Co Morb, % (SE)	26.7 (1.8)	28.5 (1.8)	26.1 (2.5)	27.3 (1.1)	47.4 (2.6)‡	36.0 (2.2)‡	29.4 (2.6)	38.0 (1.4)‡
	**Wealth**							
	**Low**	**Medium**	**High**	**Total**	**Low**	**Medium**	**High**	**Total**
*Age 50 to 64 years*								
N hypertensive¤	243	224	259	726	237	225	206	668
Prevalence, % (SE)	43.3 (2.7)	31.0 (2.3)	28.1 (2.1)	33.4 (1.8)	53.1 (2.9)‡	42.2 (2.6)‡	32.1 (2.4)	41.7 (1.7)‡
Sex (% Female, SE)¤	48.2 (3.2)	53.8 (3.4)	46.2 (3.2)	49.3 (1.9)	48.1 (4.5)	41.7 (4.7)	37.2 (4.6)	42.8 (2.7)
Age, mean (SE)¤	60.1 (0.2)	60.2 (0.2)	60.1 (0.2)	60.1 (0.1)	58.9 (0.3)	59.1 (0.3)	59.6 (0.3)	59.2 (0.2)
Current smoker, % (SE)	27.3 (2.9)	13.9 (2.4)	7.5 (1.8)	16.7 (1.5)	30.0 (3.4)	18.8 (3.0)	10.1 (2.4)	20.0 (1.8)
BMI mean (SE)	31.4 (0.4)	30.0 (0.3)	29.2 (0.3)	30.2 (0.2)	32.1 (0.6)	31.8 (0.5)	30.3 (0.5)	31.4 (0.3)‡
>1 Vascular Co Morb, % (SE)	24.4 (2.8)	13.1 (2.2)	10.0 (1.9)	16.2 (1.4)	44.9 (4.4)‡	23.6 (4.1)‡	22.6 (4.2)‡	31.4 (2.5)‡
Insurance coverage, % (SE)					83.5 (3.3)	91.9 (2.6)	97.7 (1.6)	90.5 (1.6)
*Age*≥*65 years*								
N hypertensive¤	513	594	485	1592	586	778	823	2187
Prevalence, % (SE)	58.4 (1.7)	54.1 (1.5)	47.4 (1.6)	53.4 (0.9)	67.8 (1.8)‡	65.2 (1.5)‡	58.7 (1.4)‡	63.1 (0.9)‡
Sex (% Female, SE)¤	62.6 (2.2)	57.1 (2.0)	50.0 (2.3)	57.2 (1.3)	61.1 (2.8)	47.6 (2.5)	43.4 (2.3)	49.7 (1.5)
Age, mean (SE)¤	77.2 (0.4)	76.1 (0.4)	75.1 (0.4)	76.2 (0.2)	74.5 (0.4)	74.3 (0.4)	74.4 (0.3)	74.4 (0.2)
Current smoker, % (SE)	15.3 (1.6)	6.5 (1.0)	5.1 (1.1)	9.3 (0.8)	14.8 (1.6)	9.8 (1.2)	5.7 (1.0)	9.6 (0.7)
BMI mean (SE)	29.1 (0.2)	29.0 (0.2)	28.0 (0.2)	28.8 (0.1)	29.4 (0.4)	29.9 (0.3)‡	29.6 (0.3)‡	29.7 (0.2)‡
>1 Vascular Co Morb, % (SE)	34.1 (2.1)	26.0 (1.8)	19.8 (1.9)	27.3 (1.1)	47.2 (2.9)‡	38.7 (2.5)‡	30.9 (2.2)‡	38.0 (1.4)‡

BMI  =  Body Mass Index; BP = Blood Pressure; SE = standard error; Co Morb  =  co-morbidity; Age controlled to 57 in former age group and 75 in latter.

Not tested for statistical significance controlling for age and sex.

†p<0.05.

‡p<0.01 testing the English against the US, controlling for age and sex.

### US Medicare v English NHS (> 65 years)

There was significantly higher aggregate blood pressure (BP) control in US respondents aged >65 years using clinical (BP<140/90 mm Hg- 53.5% England vs. 58.2% in US; p = 0.043) but not audit targets (BP<150/90 mm Hg- 71.9% vs. 73.9% respectively; p = 0.338) (table 2). Mean systolic BP was significantly lower in the US than England (135.6 vs. 140.2 mm Hg; p<0.001) but the converse for mean diastolic BP (78.9 vs. 73.7 mm Hg; p<0.001). Prescribing of at least one anti-hypertensive medication was significantly more common in the US than England (91.4% vs. 80.9%; p<0.001).

**Table 2:** Aggregate blood pressure control and prescribing in hypertensive patients aged 50- 64 in the United States and England.

		England	United States	
		Total	Total	Insured
Aged ≥65 years^a^	Systolic BP mean (SE)	140.2 (0.65)	135.6 (0.64)‡	–
	Diastolic BP mean (SE)	73.7 (0.36)	78.9 (0.34)‡	–
	BP <140/90 mmHg, % (SE)	53.5 (1.73)	58.2 (1.54)†	–
	BP <150/90 mmHg, % (SE)	71.9 (1.55)	73.9 (1.39)	–
	Anti-hypertensive medication, % (SE)	80.9 (1.34)	91.4 (0.91)‡	–
				
Aged 50-64 years^b^	Systolic BP mean (SE)	135.5 (1.17)	131.2 (1.05)‡	129.9 (1.09)‡
	Diastolic BP mean (SE)	81.5 (0.70)	84.8 (0.68)‡	83.9 (0.68)†
	BP <140/90 mmHg, % (SE)	61.3 (3.55)	62.3 (3.15)	65.4 (3.33)
	BP <150/90 mmHg, % (SE)	74.5 (3.24)	69.4 (3.06)	72.7 (3.15)
	Anti-hypertensive medication, % (SE)	56.4 (3.71)	80.2 (2.61)‡	84.2 (2.56)‡

†p<0.05.

‡p<0.01 testing US groups against the English total.

^a^ US Medicare v English NHS group.

^b^ US market-based v English NHS; BP = Blood Pressure; SE = standard error.

All values and significance testing controls for age and sex, BMI, vascular co-morbidity and smoking (age controlled at 75 & 57 respectively, female sex, non-smoker, no co-morbidity and BMI = 28.8 – the English mean).

The lowest SEP groups had poorer control to clinical targets in the US, which reached statistical significance for income (56.3% in low income vs. 62.9% in high; p = 0.033), but not in England (table 3). There were no statistically significant SEP disparities to audit targets. When the analysis compared equivalent SEP groups between countries (results not presented), the largest disparity in blood pressure control was present in highest SEP groups (e.g. income – lowest 53.9% in England vs. 56.3% in US, p = 0.533; highest 51.0% vs. 62.9% respectively, p = 0.033).

**Table 3:** Blood pressure control and prescribing in hypertensive patients in US Medicare v English NHS by socioeconomic-position (> 65 years).

	England			United States		
	Years of Education					
	Low	Medium	High	Low	Medium	High
Systolic BP mean (SE)	140.1 (0.73)	142.1 (3.00)	140.0 (1.11)	136.7 (0.78)	133.9 (1.24)†	134.6 (1.05)
Diastolic BP mean (SE)	73.4 (0.41)	75.6 (1.44)	74.8 (0.64)†	79.0 (0.42)	78.3 (0.65)	79.4 (0.57)
BP <140/90 mmHg, % (SE)	53.6 (1.94)	47.8 (6.30)	54.2 (3.03)	56.8 (1.90)	57.9 (2.83)	61.5 (2.67)
BP <150/90 mmHg, % (SE)	71.8 (1.76)	66.6 (6.20)	73.1 (2.79)	72.8 (1.71)	73.6 (2.51)	76.5 (2.35)
Anti-hypertensive medication, % (SE)	81.0 (1.48)	80.0 (5.16)	80.3 (2.45)	90.3 (1.21)	92.7 (1.87)	92.8 (1.54)

	Income					
	Low	Medium	High	Low	Medium	High
Systolic BP mean (SE)	139.9 (0.96)	139.9 (0.89)	141.1 (1.16)	136.8 (1.10)	136.3 (0.86)	133.7 (0.94)†
Diastolic BP mean (SE)	73.4 (0.53)	73.7 (0.51)	74.4 (0.63)	79.6 (0.58)	78.7 (0.47)	78.6 (0.50)
BP <140/90 mmHg, % (SE)	53.9 (2.49)	54.5 (2.37)	51.0 (3.04)	56.3 (2.43)	55.8 (2.12)	62.9 (2.29)†
BP <150/90 mmHg, % (SE)	72.0 (2.27)	74.3 (2.11)	67.2 (2.89)	72.4 (2.20)	71.9 (1.95)	77.5 (1.96)
Anti-hypertensive medication, % (SE)	81.7 (1.88)	80.5 (1.80)	79.9 (2.48)	89.6 (1.88)	92.7 (1.10)	91.4 (1.46)

	Wealth					
	Low	Medium	High	Low	Medium	High
Systolic BP mean (SE)	141.1 (1.10)	139.4 (0.94)	140.3 (0.93)	135.4 (1.21)	137.0 (0.93)	134.7 (0.82)
Diastolic BP mean (SE)	73.1 (0.62)	73.7 (0.52)	74.3 (0.52)	79.1 (0.65)	79.1 (0.50)	78.8 (0.45)
BP <140/90 mmHg, % (SE)	51.2 (2.87)	55.4 (2.41)	53.1 (2.50)	56.6 (2.76)	56.3 (2.21)	60.3 (1.99)
BP <150/90 mmHg, % (SE)	69.5 (2.70)	74.8 (2.15)	70.3 (2.31)	74.2 (2.46)	71.1 (2.02)	75.7 (1.74)
Anti-hypertensive medication, % (SE)	80.6 (2.16)	80.0 (1.90)	82.0 (1.96)	87.4 (2.46)	92.4 (1.23)†	92.4 (1.11)†

BP = Blood Pressure; SE = standard error; Values and significance testing controls for age and sex, body mass index, vascular co-morbidity and smoking (age controlled at 75, female sex, non-smoker, no co-morbidity and BMI = 28.8 – the English mean). Significance tests compare the medium and high SEP groups with the low within each country.

†p<0.05.

There was higher prescribing of anti-hypertensive medication in the US across all SEP groups compared with England. In England there was no SEP disparity in prescribing, whereas the US had lower prescribing in lowest SEP groups, which was statistically significant for wealth (87.4% in the least wealthy vs. 92.4% in the most; p = 0.029).

### US Market-based v English NHS (50–64 years)

In patients with hypertension aged 50–64 years, there were no significant differences in the control of blood pressure to 140/90 mm Hg between countries (table 2). Respondents with health insurance in the US had similar control to those in England (61.3 in England vs. 62.3 in all Americans (p = 0.835 vs. England) and 65.4% in the insured (p = 0.409 vs. England). Similarly there were no statistically significant differences in control to audit targets in the whole population (74.5 in England vs. 69.4 in the US; p = 0.255), or insured Americans (72.7; p = 0.695).

Despite no overall difference between countries in the 50–64 years age group, Americans in the highest wealth groups had significantly better control to the clinical target than their English counterparts (table 4) (71.7 vs. 60.9; p = 0.037). Wealth disparities to both clinical and audit targets were present in the US, with wealthier patients more likely to meet targets, but not in England (clinical target- 60.9% for wealthy vs. 63.5% for poorer patients in England (p = 0.588); compared with 71.7% vs. 55.2% (p = 0.003) in the US). The wealth disparity is reduced, but still significant in the insured, under-65 American group (72.5 in the most wealthy vs. 60.7 in the least (p = 0.036)). There was a suggestion of an income disparity in the US sample, with the intermediate group having better control than the lowest (clinical target – 54.9% in low income vs. 66.6% in intermediate (p = 0.05)). This was not, however statistically significant for the highest income (62.2%; p = 0.198). There were no income disparities in the English sample.

**Table 4:** Blood pressure control and prescribing in hypertensive patients in US market-based v English NHS by socioeconomic-position (aged 50-64 years).

	England			United States					
	Total			Total			Insured		
	Years of Education								
	Low	Medium	High	Low	Medium	High	Low	Medium	High
Systolic BP mean (SE)	136.9 (1.35)	131.3 (2.10)‡	134.2 (1.46)	132.8 (1.42)	131.5 (1.70)	129.0 (1.45)†	131.2 (1.50)	130.6 (1.76)	128.0 (1.46)
Diastolic BP mean (SE)	81.9 (0.82)	81.0 (1.47)	81.1 (0.85)	84.5 (0.93)	85.0 (1.04)	84.9 (0.95)	83.4 (0.98)	84.3 (1.00)	84.0 (0.93)
BP <140/90 mmHg, % (SE)	57.8 (4.17)	70.3 (6.52)	64.6 (4.30)	59.3 (4.18)	59.7 (4.80)	68.0 (4.20)	63.2 (4.46)	62.5 (5.00)	70.1 (4.28)
BP <150/90 mmHg, % (SE)	71.9 (3.89)	76.6 (6.15)	78.2 (3.71)	67.4 (4.09)	70.2 (4.61)	70.9 (4.21)	71.7 (4.22)	72.4 (4.64)	73.8 (4.22)
Anti-hypertensive medication, % (SE)	53.4 (4.32)	58.0 (7.06)	61.3 (4.58)	78.4 (3.89)	77.4 (4.23)	84.4 (3.50)	84.3 (3.87)	80.2 (4.01)	87.2 (3.32)

	Income								
	Low	Medium	High	Low	Medium	High	Low	Medium	High
Systolic BP mean (SE)	134.0 (1.62)	136.3 (1.61)	135.7 (1.35)	135.5 (1.87)	129.5 (1.63)‡	130.9 (1.22)†	132.6 (2.09)	128.2 (1.70)	130.2 (1.24)
Diastolic BP mean (SE)	81.4 (0.97)	81.7 (0.97)	81.5 (0.82)	86.5 (1.18)	83.3 (1.09)†	85.1 (0.75)	84.4 (1.32)	82.3 (1.13)	84.4 (0.73)
BP <140/90 mmHg, % (SE)	62.6 (4.95)	58.1 (4.77)	62.6 (4.01)	54.9 (5.67)	66.6 (4.42)†	62.2 (3.60)	62.4 (6.38)	69.1 (4.58)	64.5 (3.73)
BP <150/90 mmHg, % (SE)	75.3 (4.52)	74.2 (4.24)	74.2 (3.68)	62.5 (5.82)	75.8 (3.94)†	68.2 (3.55)	71.4 (6.25)	78.6 (3.91)	70.5 (3.61)
Anti-hypertensive medication, % (SE)	51.7 (5.41)	61.6 (4.59)	55.6 (4.35)	74.9 (5.38)	83.8 (3.77)	80.0 (3.08)	81.4 (5.34)	89.3 (3.25)	82.6 (3.06)

	Wealth								
	Low	Medium	High	Low	Medium	High	Low	Medium	High
Systolic BP mean (SE)	133.7 (1.59)	135.9 (1.45)	136.4 (1.49)	133.8 (1.62)	131.9 (1.58)	128.6 (1.29)†	131.6 (1.73)	130.7 (1.65)	128.2 (1.33)
Diastolic BP mean (SE)	81.1 (0.95)	81.0 (0.87)	82.2 (0.90)	85.6 (1.14)	85.5 (0.95)	83.5 (0.80)	83.9 (1.18)	84.6 (0.98)	83.2 (0.81)
BP <140/90 mmHg, % (SE)	63.5 (4.61)	59.7 (4.61)	60.9 (4.37)	55.2 (4.74)	57.6 (4.39)	71.7 (3.95)‡	60.7 (5.07)	60.4 (4.67)	72.5 (4.08)†
BP <150/90 mmHg, % (SE)	75.1 (4.34)	74.4 (4.04)	74.0 (3.96)	64.3 (4.84)	64.2 (4.36)	78.0 (3.51)‡	69.3 (4.99)	67.6 (4.57)	78.9 (3.56)
Anti-hypertensive medication, % (SE)	57.1 (5.04)	59.3 (4.64)	53.8 (4.53)	71.2 (5.18)	80.5 (3.67)	86.0 (2.83)‡	81.6 (4.70)	81.6 (3.86)	87.7 (2.73)

BP = Blood Pressure; SE = standard error; Values and significance testing control for age and sex, body mass index, vascular co-morbidity and smoking (age controlled at 57, female sex, non-smoker, no co-morbidity and BMI = 30.2 – the English mean in the age group. Significance tests compare the medium and high SEP groups with the low within each country.

†p<0.05.

‡p<0.01.

Mean systolic blood pressure was lower in the total and insured American groups aged under 65 years compared with the sample from England (England = 135.5; insured American = 129.9 (p<0.001 vs. England); total American = 131.2 (p = 0.007 vs. England)): Diastolic blood pressure was lower in the English sample, compared with both from the US (England = 81.5; insured American = 83.9 (p<0.016 vs. England); total American = 84.8 (p<0.001 vs. England)).

English patients with hypertension were less often prescribed anti-hypertensive medication than insured Americans and Americans in total (56.4%, 84.2% (p<0.001 vs. England) and 80.2% (p<0.001 vs. England), respectively). There were no SEP disparities in prescribing in the US Sample, but there was statistically significantly lower prescribing in the lowest wealth group in the total US sample compared with the lowest (71.2% in lowest vs. 86.0% in the highest; p = 0.004). There were no prescribing disparities in the US sample reporting health insurance.

## Discussion

### Main findings

Patients with hypertension in America eligible for Medicare are, overall, more likely than their English counterparts to have their blood pressure controlled to the clinical guideline target, but not to the English audit target. Patients aged 50 to 64 years, with insurance coverage through the NHS or under the market-based US health system, have similar control to both targets, even limiting the comparison to US patients reporting health insurance. The US has significantly more prescribing of anti-hypertensive medication across all levels of SEP. We found no evidence of disparities in English patients, while significant wealth and income disparities in blood pressure control and prescribing among younger US patients. Disparities in both were generally attenuated but not abolished in insured patients aged under-65 and older patients eligible for Medicare.

The US is enacting some of its most significant health care reforms since the formation of Medicare in 1965 [22]. Currently, up to 46 million Americans are uninsured [22], with gaps in health insurance estimated to account for up to 45,000 deaths per year [23]. The ACA aims to extend health insurance coverage to an additional 31 million citizens. An open question is whether access to health insurance will improve disease management and reduce health disparities.

While health insurance may reduce health disparities through improved access to care, the US and UK health systems differ in other ways. Primary care plays a significantly greater role in England, which itself may reduce disparities [7], [24]. Importantly, we not only saw reduced disparities in England compared with the US, but also in American patients eligible for Medicare compared with younger patients in the US. Insurance coverage may be a significant driver of disparities in hypertension control in younger Americans. In the US, younger patients with market-based (often private) insurance may trade insurance source (switching to the Medicare) once eligible. Younger patients lacking insurance (usually from a lower SEP) are more likely to become newly eligible for insurance at the age of 65 years [25] and in turn see greatest increases in health care expenditure [26]. This might, in part, reduce US disparities in hypertension management.

That said, disparities in blood pressure control are absent in older English patients but some remain in Medicare-eligible Americans (for example an income disparity to clinical blood pressure targets). Universal coverage alone may not eliminate disparities. The strength of English primary care may increase equity in hypertension control [7]. Also, in the US, even Medicare patients, and many with private insurance, face significant co-payments. These may stop patients obtaining necessary care, including office visits and pharmaceuticals. Up to 40 percent of patients with chronic conditions in the US report significant out-of-pocket payments (>$1,000), compared with only 4 percent in the UK [27]. Moves under the ACA not only to extend health insurance but also to impose minimum quality standards on all policies, including ending annual claims limits and eliminating certain co-payments, may limit obstacles to appropriate care for Americans, and improve equity in care [13]. Alternately, inequalities formed under the age of 65 years may simply become too entrenched to be fully eradicated under Medicare. SEP may be a *fundamental cause* of raised blood pressure [28]; however, health systems should still seek to provide equitable management for those with diagnosed hypertension [29].

In addition to more equitable care, the NHS matches the US in absolute levels of blood pressure control in the sample aged 50 to 64 years. Given greater health care spending in the US [10], this may suggest more efficient hypertension management under the NHS than in a US system with no mandated health insurance Reasons for this may include increased access to primary care, less overcharging, less duplication of care, less overuse and lower transaction/overhead costs [10], [30]. Moreover, in the US system, patients – even those with health insurance – are considerably more likely to forgo necessary care due to cost and less likely to have a long-term relationship with a health care provider [27]. Patients in the US eligible for Medicare do, however, see better control, than the sample aged ≥65 years under the NHS. This supports previous findings of greater blood pressure control in patients with hypertension in the US than in England [31]. The US may have improved treatment strategies, including the medication use seen here. Systolic blood pressure was lower in the US sample, but diastolic blood pressure significantly lower in England (up to a 5 mm. Hg. difference aged ≥65). Diastolic blood pressure may be more easily controlled with medication than systolic blood pressure [32].

One interesting finding is that anti-hypertensive medications are more far more frequently prescribed in the US sample aged 50 to 64 years, but control remains comparable. The control of blood pressure in hypertensive patients is not solely determined by the prescribing of pharmacological agents *per se*. Other factors, including use of appropriate combination therapy and life-style interventions, are important [33]. This might, at least in part, explain the lack of association between prescribing rates and blood pressure control. This may be especially relevant in a US sample with no single insurance source, which is exposed to under-insurance and co-payment [27]. Our data did not permit us to determine which medications were prescribed (nor how many). Therefore, we could not assess the appropriateness of prescribing (including combination therapy).

### Strengths & Limitations

Our study has potential limitations. We used patient-reported diagnoses of hypertension. While this definition may underestimate prevalence [5], we felt it more appropriate than one based on blood pressure values, which will include undiagnosed cases. After all, the health system is only able to manage disease when diagnosed and recorded. Use of self-reporting may have influenced our findings. Most notably, we may underestimate levels of prescribing, although differences between countries are likely to be minimal. We have data on only one aspect of hypertension management; anti-hypertensive prescribing, however, this is a core aspect of control, highlighted by clinical guidelines in both countries. We did not have data on the class of antihypertensive agent prescribed, or the number. These differences may contribute to the cross-national differences in blood pressure control, and may require further study. While hypertension is a common, high burden disease and our study provides key insights related to its management, it may not be appropriate to extrapolate findings to other disease areas, particularly higher cost acute conditions. We limited our study to white respondents in each country. This allowed us to focus on national differences in health care insurance and policies, without confounding from ethnically different minority groups. This allows greater focus on the research question, improves validity, but will limit the applicability of our findings to the general population in both countries. Our data only cover older adults, so findings may not be generalisable to all ages. Finally, the survey contained missing data for outcomes and covariates. The nested survey design, however, combined with the weights, ensure those with complete data remain representative.

### Impacts on policy and practice

Hypertension control in both countries remains suboptimal; blood pressure is controlled to clinical targets in just over a half of patients aged 65 years and over. Given the association between blood pressure control and good health outcomes [34], and despite an array of efficacious medications [3], further efforts are required to improve the management of hypertension in both countries. Chronic disease control may also be influenced by population factors, including dietary behaviours and lifestyle [35]. Managing underlying causes of disease and poor control warrant greater attention [36].

The English and American health care systems differ in many ways, including the provision of universal coverage and overall levels of expenditure. Our study demonstrates that hypertension control in England matches that in the US in a sample without universal health insurance, despite the evidence elsewhere of lower overall expenditure. The two study samples covered by universal health insurance had the lowest disparities in hypertension control, and in one – in England – were entirely absent. The ACA's vision of improving access to healthcare may promote equitable care. Interestingly, while there were no indications of health care disparities in the English system, some persisted in the US, even when patients had insurance (either private insurance for patients under 65 years or Medicare for older patients). The introduction of universal coverage at the age of 65 years may not eliminate disparities that become too entrenched; although may reduce them. Again, the ACA's expansion of Medicare and its individual mandate – expanding private insurance coverage – may increase equity. Alternately, other country-level differences, such as the underlying structures or subtle challenges to care access, including co-payment, may perpetuate disparities: If so, further system-level changes may be required, which the ACA only begins to tackle.

## Conclusions

Characteristics of both a single-payer health service and universal health insurance coverage may facilitate high-quality, equitable treatment. The expansion of Medicaid eligibility and widening private insurance coverage under the ACA, indeed the promotion of universal health coverage globally [37], may encourage equitable management of chronic conditions such as hypertension. Unlike disparities in disease prevalence which appear influenced by, amongst other things, wider redistributive social policy and the prevalence of disease risk factors [5], [6], our findings suggest disparities in hypertension control may be more closely linked to health system structure, notably access to care through insurance. Although our study demonstrates that not all kinds of “universal” health insurance are alike, it supports the ambition that expanding health insurance may reduce health disparities.
